# Predictive power of the DSM-5 criteria for internet use disorder: A CHAID decision-tree analysis

**DOI:** 10.3389/fpsyg.2023.1129769

**Published:** 2023-02-22

**Authors:** Laura Bottel, Matthias Brand, Jan Dieris-Hirche, Magdalena Pape, Stephan Herpertz, Bert Theodor te Wildt

**Affiliations:** ^1^LWL-University Hospital, Department of Psychosomatic Medicine and Psychotherapy, Ruhr University Bochum, Bochum, Germany; ^2^General Psychology: Cognition and Center for Behavioral Addiction Research (CeBAR), University of Duisburg-Essen, Duisburg, Germany; ^3^Erwin L. Hahn Institute for Magnetic Resonance Imaging, Essen, Germany; ^4^Psychosomatic Hospital Diessen Monastery, Diessen am Ammersee, Germany

**Keywords:** internet use disorder, diagnostic criteria, DSM-5, ICD-11, gaming disorder, internet gaming disorder, CHAID, decision tree analysis

## Abstract

**Introduction:**

Although the majority of internet users enjoy the internet as a recreational activity, some individuals report problematic internet use behaviors causing negative psychosocial consequences. Therefore, it is important to have precise and valid diagnostic criteria to ensure suitable treatment for those affected and avoid over-pathologization.

**Methods:**

The aim of the present study was to determine which of the nine DSM-5 criteria of internet gaming disorder (IGD) are crucial in distinguish pathological from non-pathological internet use based on the questionnaire-based response behavior of the participants by applying the Chi-squared automatic interaction detection (CHAID) decision tree analysis. Under consideration of the nine DSM-5 criteria for IGD and according to the short-form scale to assess Internet Gaming Disorder (IGDS-SF9) the DSM-5 criteria were formulated as questions and applied to the broader concept of Internet Use Disorder (IUD). The nine questions were answered on a 5-point Likert scale from “never” to “very often.” In accordance with the IGDS-SF9 participants were assigned to IUD-5plus if at least 5 of the 9 criteria were answered with “very often.” The study was conducted in Germany (*N* = 37,008; *mean age*: 32 years, *SD* = 13.18, 73.8% male).

**Results:**

Although “loss of control,” “continued overuse” and “mood regulation” were the most endorsed criteria, the analysis indicated that the criterion “jeopardizing” was found as the best predictor for IUD-5plus, followed by “loss of interest” and “continued overuse.” Overall 64.9% of all participants who were in the IUD-5plus, could been identified by the fulfillment of the three criteria mentioned above.

**Discussion:**

The results found support for adjustment of the DSM-5 criteria of IGD in accordance to ICD-11. If the predictive power of the three criteria can be replicated in future representative studies, such a decision tree can be used as guidance for diagnostics to capture the particularly relevant criteria.

## 1. Introduction

Although the majority of internet users enjoy the internet as a recreational activity and use internet applications in a functional manner, some individuals report uncontrolled and problematic internet use behaviors that results in negative psychosocial consequences (Rumpf et al., 2018; Brand, 2022). According to epidemiological studies, an average of 7.02% of individuals worldwide are affected by (unspecified) internet use disorders (IUD; Pan et al., 2020) and 1.96–3.05% of adolescents by gaming disorder (GD; Stevens et al., 2021), with increasing prevalence for younger age groups (Rumpf et al., 2014; Pan et al., 2020).

Building on the large number of studies on GD, the American Psychiatric Association included internet gaming disorder (IGD) as a specific type of IUD in the research appendix of the fifth edition of the Diagnostic and Statistical Manual for Mental Disorders (DSM-5; American Psychatric Association, 2013). In 2018 GD has then been included in the 2022 published International Statistical Classification of Diseases and Related Health Problems (ICD-11) by the World Health Organization within the category “disorders due to addictive behaviors” (World Health Organization, 2022b).

In addition to IGD, there is growing evidence that other internet activities such as excessive use of internet pornography, online shopping or the use of social networking sites can lead to similar addictive behavioral patterns and may be considered as “other specific disorders due to addictive behavior” in the ICD-11 under the condition that functional impairment is present (Brand et al., 2020, 2022). Therefore, IUD is used as an umbrella term for several subtypes (Montag et al., 2021). Furthermore Müller and colleagues were able to demonstrate in a clinical study, that no particular differences were found for the applicability of IGD criteria to IUD (Müller et al., 2019). Based on this and following the approach of an epidemiological study in Germany (Bischof et al., 2013), the DSM-5 criteria for IGD were related to the broader concept of IUD in the present study.

According to the DSM-5, a diagnosis of IGD is given when five (or more) of the following nine criteria are met: (1) preoccupation with internet games, (2) withdrawal symptoms when internet gaming is taken away, (3) tolerance – the need to spend increasing amounts of time engaged in internet games, (4) loss of control - unsuccessful attempts to control the participation in internet games, (5) loss of interests in previous hobbies and entertainment as a result of, and with the exception of, internet games, (6) continued overuse – excessive use of internet games despite knowledge of psychosocial problems, (7) deceived family members, therapists or others regarding the amount of internet gaming, (8) mood regulation – use of internet games to escape or relieve a negative mood (e.g., feelings of helplessness, guilt, anxiety), (9) jeopardized or loss of a significant relationship, job or educational or career opportunity because of participation in internet games (American Psychatric Association, 2013; Petry et al., 2015). The inclusion of IGD in the DSM-5 in 2013 provided standardized diagnostic criteria for this relatively new disorder as a guidance for research and practice. The validity of the specific criteria, however, is still discussed controversially (Castro-Calvo et al., 2021).

A prevalence study conducted in Germany with adolescents showed that “loss of interest” (called “give up other activities” in the respective paper) and “tolerance” were the best predictors of IGD and that “mood regulation” (called “escape adverse moods” in the respective paper) and “preoccupation” were less likely to predict IGD (Rehbein et al., 2015). Results from a study of online gamers (average age 22 years) in Hungary showed that “preoccupation” and “mood regulation” (called “escape” in the respective paper) provided very little information to the estimation of IGD severity (Király et al., 2017). These findings are in line with Besser et al. (2019), who identified that the “mood regulation” criterion (called “escape from a negative mood” in the respective paper) may be insufficient to distinguish between problematic and non-problematic internet use. A survey of nearly 30,000 students in China indicated that “loss of interest” (called “give up other activities” in the respective paper), “jeopardizing” (called “negative consequences” in the respective paper) and “continued overuse” (called “continue despite problems” in the respective paper) best predict a diagnosis of IGD (Luo et al., 2021).

Although the validity of specific DSM-5 criteria has been demonstrated in studies across different cultures with divers findings, there are only a few clinical studies, in which the IGD diagnostic criteria have been investigated. In those few studies that applied the criteria in a clinical setting satisfactory diagnostic validity of specific DSM-5 criteria was shown, whereas the criteria “mood regulation” (called “escape” in the respective paper) and “deception” had lower diagnostic validity (diagnostic accuracy <80%; Ko et al., 2014; Müller et al., 2019). The DSM-5 diagnostic criteria of IGD are close to those of disorders due to substance use (World Health Organization, 2022a), which was criticized and there is a growing consensus that certain criteria (e.g., tolerance, preoccupation) cannot be applied to such behaviors and may lead to inappropriate diagnosis and over-pathologization (Kass, 1980; Kardefelt-Winther, 2015; Starcevic, 2016; Billieux et al., 2019).

In contrast to the DSM-5 criteria, GD is defined as behavioral addiction in the ICD-11 and the criteria are defined by only three core criteria: (1) impaired control over gaming behaviors, (2) increasing priority of gaming to the extent that gaming takes precedence over other life interests and daily activities, (3) continuation or escalation of gaming despite the occurrence of negative consequences. In addition, the gaming behavior must result in marked distress or significant impairments in important areas of functioning to justify the diagnosis of GD (World Health Organization, 2022b). Contrary to the DSM-5 criteria, all of the above-mentioned ICD-11 criteria must be present to diagnose a GD. Besides the required ICD-11 criteria to diagnose GD, additional clinical features such as increase of duration or frequency of gaming behavior, cravings to engage in gaming during other activities and/or substantial disruptions in diet, sleep, exercise and other health-related behaviors that result in negative physical and mental health outcomes were listed in the ICD-11. These additional clinical features outline further potential characteristics of this disorder, but the features are not essential for the diagnosis of GD (World Health Organization, 2022b).

In an initial study comparing the DSM-5 criteria and ICD-11 criteria among high-risk adolescents in Korea, 32.4% of participants met the DSM-5 criteria, whereas only 6.4% of the same sample met ICD-11 criteria (Jo et al., 2019). Although evidence for the ICD-11 criteria is currently lacking, these results already suggest that the strict ICD-11 criteria of GD may prevent false positive diagnoses (Billieux et al., 2017, 2019; Jo et al., 2019). These results are consistent with an international Delphi-study, in which an expert panel of scientific and/or clinical experts in the field of GD concluded that “mood regulation” (called “escapism/mood regulation” in the respective paper) and “tolerance” as diagnostic criteria were incapable of distinguishing between problematic and non-problematic gaming (Castro-Calvo et al., 2021). Based on the expert panel, all ICD-11 criteria for GD were judged as presenting high diagnostic validity, clinical utility and prognostic value. Whether these criteria may be useful to diagnose other types of IUD or even unspecified IUD is unclear so far.

Thus, there seem to be similarities, but also significant differences between the two classification systems for diagnosis of (I)GD. In order to achieve a common understanding of the disorder and the diagnostic criteria, a detailed review of the criteria and comparison is essential. A growing number of researchers and practitioners are encouraging to differentiate between core symptoms and motivations, mechanisms and processes, in order to distinguish between IUD and non-pathological use (Billieux et al., 2019; Brand et al., 2020).

Due to the high discrepancy between the number of recreational internet users worldwide and individuals who use the internet to a pathological extent with significant negative consequences in their daily life and under consideration of the existing different nosological classifications it is important to have precise and valid diagnostic criteria to ensure suitable treatment for those affected and avoid over-pathologization. In order to capture all subtypes of an IUD and following a large-scale German epidemiological study (Bischof et al., 2013) together with a study which showed that no particular differences were found for the applicability of the IGD criteria to IUD (Müller et al., 2019), the aim of the present study was to analyze which of the nine DSM-5 criteria of IGD are crucial in distinguishing between IUD and unproblematic internet use using a large dataset with over 37,000 participants.

## 2. Methods

### 2.1. Participants and procedure

Between September 2016 and December 2019, the telemedicine study “Online-Ambulatory Service for Individuals with Internet Use Disorder and their Relatives” (OASIS; Bottel et al., 2021) was conducted in Germany and funded by the German Federal Ministry of Health (ZMVI1-2516DSM207). The self-test represented the first part of the OASIS project.

The intention was to create a freely accessible, low-threshold offer for interested persons with direct feedback regarding their own internet use. Interested persons had the opportunity to anonymously fill out an online questionnaire (self-test) assessing their internet use *via* the project homepage. Immediately after completing the questionnaire, participants received feedback regarding their usage behavior and whether participation in the OASIS-project was recommended.

Recruitment took place at various levels. The cooperation partner Fachverband Medienabhängigkeit e.V. (largest German association regarding IUD) drew attention to the project and the online self-test through its network of professionals, (former) person affected, relatives and interested person. At the same time, media interest in the project and the topic of IUD was very high, resulting in numerous newspaper articles, blog posts, interviews on radio and television about the project and the possibility of a freely available online self-test. Furthermore, the project had its own social media account and was presented with its own stand at the largest computer game convention, where interested person had the opportunity to fill out the self-test on site. Recruitment was further supported by project-presentations in schools, companies, as well as presentations at congresses and in treatment institutions. Based on the answers in the self-test, two groups were defined. The first group was defined as “IUD-5plus” with participants who answered five or more of the nine questions representing the DSM-5 criteria with “very often.” The second group “IUD-4minus” was defined by participants who answered four or less questions of the nine questions representing the DSM-5 criteria with “very often.”

### 2.2. Measures

#### 2.2.1. Assessment of IUD according to DSM-5

Under consideration of the nine DSM-5 criteria for IGD and according to the short-form scale to assess Internet Gaming Disorder (IGDS-SF9; Pontes and Griffiths, 2015) the DSM-5 criteria were formulated as questions and applied to the broader concept of IUD (“gaming” was replaced by “internet activities”). Following a German epidemiological study (Bischof et al., 2013) IUD was captured instead of IGD to provide the opportunity of an online self-test for as many people as possible and since no particular differences were found for the applicability of IGD criteria to IUD in a clinical study (Müller et al., 2019). The nine questions were answered on a 5-point Likert scale from “never” to “very often.” In line with the DSM-5 guidelines to determine IGD and following the cut-offs of the IGDS-SF9 (Pontes and Griffiths, 2015), individuals were assigned to the IUD-5plus group, if five (or more) questions were answered with “very often.” The nine questions representing the DSM-5 criteria in German and the English translation can be found in the Supplementary material.

#### 2.2.2. Sociodemographic

In addition to age and gender, the federal state in which the persons in Germany lived, were recorded.

Since the study aim was to determine which of the DSM-5 criteria distinguish between pathological and non-pathological internet use based on the questionnaire-based response behavior of the participants, only the most important data to reach this aim were collected.

#### 2.2.3. Statistical analysis

Descriptive statistical analyses were calculated to describe the study population. Percentages were used for categorical variables and means and standard deviation for continuous variables. Independent-sample t-tests (2-sided) were conducted to compare the group of “IUD-4minus” with “IUD-5plus” and Chi2-tests for nominal variables. As effect size for Chi2-test the Cramer’s V was calculated with *V* = 0.1 indicating small, *V* = 0.3 medium and *V* = 0.5 large effects and for the t-tests with Cohen’s *d* indicating *d* = 0.2 small; *d* = 0.5 medium; *d* = 0.8 large – effects (Cohen, 1988, 2013). Median split was used to include the variable “age” as a dichotomous variable in the decision tree model [younger age group: 18–29 years (*n* = 19,125) and older age group: 30–79 years (*n* = 17,883)].

Chi-squared automatic interaction detection (CHAID) algorithm (Kass, 1980; Song and Ying, 2015) was used to perform the decision tree analyses with the response variable IUD-5plus. Predictive variables were age (median split younger/older age group), gender and the nine DSM-5 criteria of IGD modified for IUD. Multiple contingency tables between the dependent and each independent variable were created and the most significant Chi-squared independent variable was selected to branch out the decision tree. To avoid overfitting, the decision tree was set to have a maximum of 3 levels and a significance level was set at ≤0.05 (IBM, 2022). The cross-validation was used as decision tree validation method. The original study cohort was randomly assigned to 10 subsets of equal sizes. The cross-validation process was repeated 10 times with the same procedure for all subsets. The first tree was calculated on all datasets except those in the first subset, the second tree was calculated on all datasets except those in the second subset and so on. Every subset was only used once and the cross-validated risk for the final tree was calculated based on the average value of the 10 results (IBM, 2022).

In the course of the decision tree analysis, the criterion was classified as “applicable” if the respective question was answered with “very often” by the participants. The criterion was classified as “not applicable” if the respective question was answered with “never,” “rarely,” “sometimes” or “often” by the participants.

A careless responder analysis was conducted to identify unrealistic values in age data and, based on this, 262 participants were excluded from the analysis.

Statistical analysis were performed and figures build using IBM SPSS Statistics for Windows, version 26.0.

#### 2.2.4. Ethics statement

The study was carried out in accordance with the Declaration of Helsinki. The Institutional Review Board approved the study (16–5,734) and participants were informed about the study procedure and consented.

## 3. Results

### 3.1. Sample characteristics

Based on participants’ self-report, 3.2% of the sample met the criteria for an IUD-5plus which means that they have answered five or more of the nine questions, capturing the DSM-5 criteria, with “very often” (see Table 1). The demographic characteristics of the two groups IUD-4minus and IUD-5plus are shown in Table 1. Participants in the IUD-5plus group were on average 3 years younger and the proportion of males in this group was two percentage points lower than in the IUD-4minus group. In general, there was a significantly higher proportion of male than female participants with 73.9% male participants and an average age of 32 years (*SD* = 13.20, *range* 18–79 years) in the entire sample. Based on the median split two groups of younger- and older age were defined. Bonferroni-adjusted post-hoc tests reveal that in the younger age group the proportion of persons assigned to the IUD-5 plus group is significantly higher (4.1%) than in the older age group (2.3%).

**Table 1:** Comparison of the two groups IUD-4minus and IUD-5plus regarding sex and age for the entire sample and the two subgroups younger- and older age group based on mediansplit.

	IUD-4minus	IUD-5plus	Statistics
Entire sample (*N* = 37,008)			
Male gender – *% (n)*	73.9 (26,465)	71.1 (853)	*Chi^2^*(2) = 283.565, *p* < 0.001, *V* = 0.088
Age – *M (SD)*	32.34 (13.20)	28.81 (12.26)	*T*(37,006) = 9.127, *p* < 0.001, *d* = 0.277
Subgroups based on mediansplit			
Younger age group (18–29 years; *n* = 19,125) – *% (n)*	95.9 (18,342)	4.1 (783)	*Chi^2^*(1) = 92.137, *p* < 0.001, *V* = 0.050
Older age group (30–79 years; *n* = 17,883) – *% (n)*	97.7 (17,467)	2.3 (416)	

### 3.2. Endorsement rates

As shown in Table 2, the three criteria “loss of control” (entire sample: 9.6%, IUD-4minus: 7.0%, IUD-5plus: 88.1%), “continued overuse” (entire sample: 8.2%, IUD-4minus: 5.4%, IUD-5plus: 92.0%) and “mood regulation” (entire sample: 6.6%, IUD-4minus: 4.0%, IUD-5plus: 84.0%) were the three most frequently endorsed criteria (answered with “very often”) across the entire sample and the two subgroups IUD-4minus and IUD-5plus. The most rarely endorsed criteria were “tolerance “(entire sample: 3.7%, IUD-4minus: 1.6%, IUD-5plus: 66.2%) and “withdrawal” (entire sample: 3.7%, IUD-4minus: 1.6%, IUD-5plus: 66.8%). The criteria “deceiving” was among the three most rarely endorsed criteria (4.4%) in the entire sample, “jeopardizing” in the IUD-4minus (1.9%) and “preoccupation” in the IUD-5plus subsample (69.5%).

**Table 2:** Endorsement rates (answered question with “very often”) of the DSM-5 criteria separately for the entire sample, IUD-4minus and IUD-5plus.

Criterion	Endorsement rates					
	Entire sample (*N* = 37,008)		IUD-4minus (*n* = 35,809)		IUD-4minus (*n* = 1,199)	
	*%*	*n*	*%*	*n*	*%*	*n*
Tolerance	3.7	1,378	1.6	584	66.2	794
Withdrawal	3.7	1,384	1.6	583	66.8	801
Deceiving	4.4	1,613	2.1	748	73.6	883
Jeopardizing	4.4	1,631	1.9	671	80.1	960
Loss of interest	5.2	1,921	2.6	921	83.4	1,000
Preoccupation	5.2	1,922	3.0	1,089	69.5	833
Mood regulation	6.6	2,442	4.0	1,435	84.0	1,007
Continued overuse	8.2	3,019	5.4	1,916	92.0	1,103
Loss of control	9.6	3,570	7.0	2,514	88.1	1,056

### 3.3. CHAID decision tree analysis

**Figure 1:**
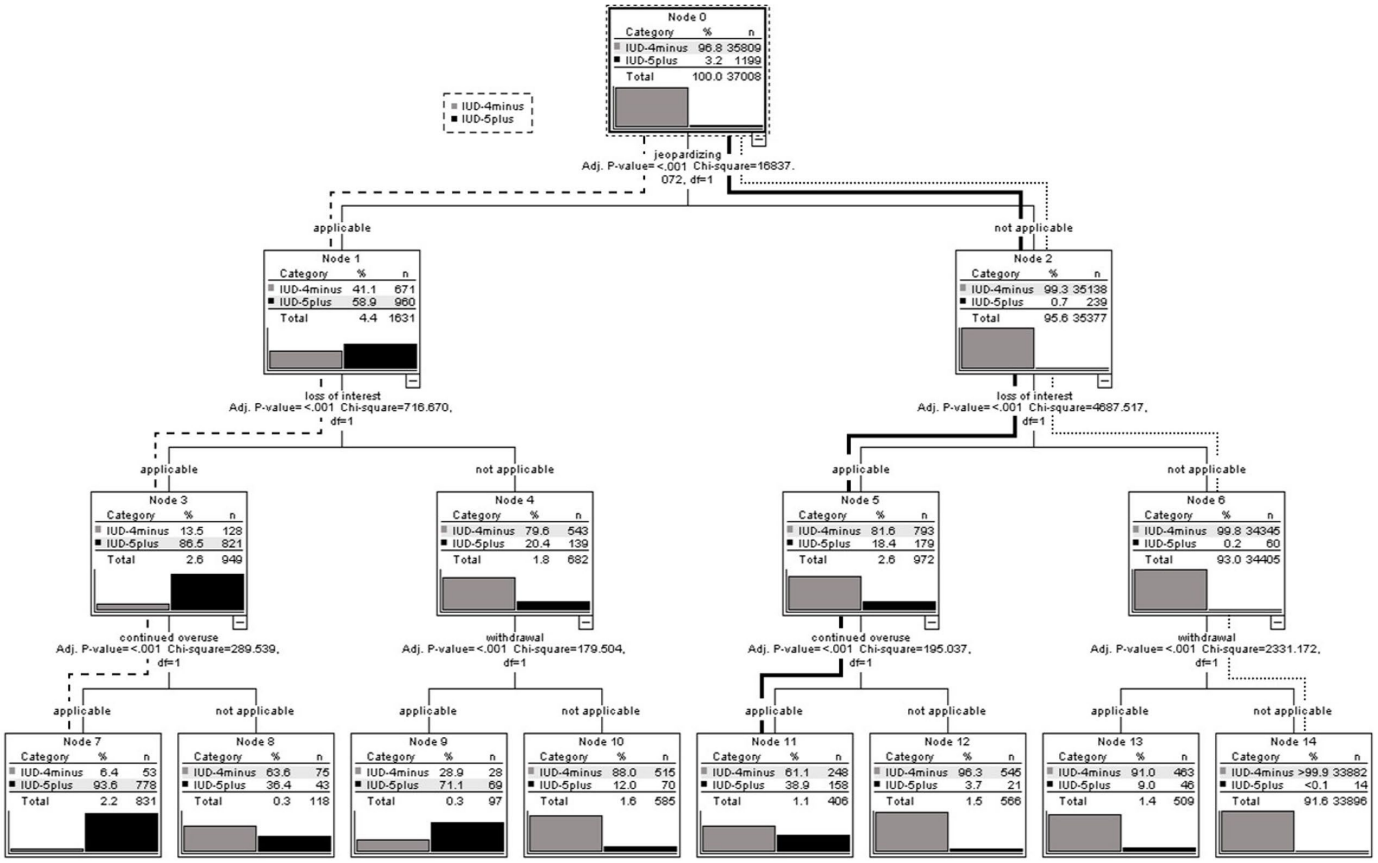
CHAID decision tree analysis with the predictive variables: gender, age (median split: younger/older age group) and the nine DSM-5 criteria for IGD on the risk of IUD-5plus for persons who were interested to receive feedback regarding their internet use behavior based on their questionnaire-based response behavior.

In the decision tree analysis, the three best predictive variables for IUD-5plus, meaning that the participants answered five or more of the nine DSM-5 criteria with “very often,” were “jeopardizing “, “loss of interest” and “continued overuse.” The model concluded with a total of eight subgroups and the overall accuracy of the model was 98.8% (see Figure 1).

To improve the comprehensibility of the results, three paths (dashed, dotted and bold line) are described in more detail below.

#### 3.3.1. Dashed line

The dashed line in Figure 1 shows the path from the first splitting variable to node 7, which subsample is defined by the highest percentage proportion of participants who fulfilled five or more out of nine DSM-5 criteria (IUD-5plus).

As seen in node 0 the criterion “jeopardizing “was defined as the first splitting variable based on the highest Chi2 value to predict IUD-5plus. The risk to be assigned to the IUD-5plus group, meaning that five or more out of nine DSM-5 criteria were answered with “very often,” was 58.9% (*n* = 960 IUD-5plus/*n* = 1,631 subsample of node 1) for the subsample of node 1. Following the dashed line in Figure 1, those participants who answered the criterion “jeopardizing” with “very often” and therefore the criterion was applicable, the criterion “loss of interest” was statistically determined as the second splitting variable with the highest predictive value within this subsample (node 1). If the second criterion “loss of interest” was applicable (answered with “very often”) within this subsample of node 1, the risk to be assigned to the group of participants who fulfilled five or more out of nine DSM-5 criteria (IUD-5plus), was 86.5% (see node 3; *n* = 821 IUD-5plus/*n* = 949 subsample node 3). Within the subsample of node 3, the third splitting variable with the highest predictive value based on the respective Chi2 values for IUD-5plus, was “continued overuse.” Among the subsample of node 7 with participants who fulfilled the three criteria “jeopardizing,” “loss of interest” and “continued overuse” (node 7), the risk of IUD-5plus based on their questionnaire-based answers was 93.6% (see node 7; *n* = 778 IUD-5plus/*n* = 831 subsample node 7).

In relation to the total number of participants who fulfilled five or more out of nine DSM-5 criteria (*n* = 1,199 IUD-5plus), 64.9% (*n* = 778 IUD-5plus in node 7/*n* = 1,199 IUD-5plus entire sample) of all participants who answered “very often” to five or more of the nine DSM-5 criteria were already been identified by the fulfillment of the criteria “jeopardizing,” “loss of interest” and “continued overuse.”

#### 3.3.2. Dotted line

In contrast to the dashed line, the dotted line in Figure 1 shows the path to node 14, which contains the lowest proportion of participants who answered five or more of the nine DSM-5 criteria with “very often” (IUD-5plus). Following the dotted line, it can be seen that if the criteria “jeopardizing,” “loss of interest” and “withdrawal” were not applicable (answered with “never,” “rarely,” “sometimes,” “often”) the risk of being assigned to the IUD-5plus group based on the questionnaire-based answers within the subsample of node 14 was 0.0004% (*n* = 14 IUD-5plus/*n* = 33,896 subsample of node 14).

#### 3.3.3. Bold line

Within the 8 subgroups at the end of the decision tree, the second highest number of participants who answered five or more out of nine DSM-5 criteria with “very often” (IUD-5plus) was found in node 11 (bold line).

Following the bold line, it can be seen, that if the criterion “jeopardizing” was not applicable, the risk of being assigned to IUD-5plus, meaning five or more out of nine criteria were answered with “very often,” was 0.7% (see node 2). Within this subsample of node 2 the criterion with the highest predictive value to identify participants who answered five or more out of nine DSM-5 criteria with “very often” (IUD-5plus) based on the Chi2 values, was “loss of interest.” Following the bold line to node 5 it can be seen, that if the criterion “loss of interest” was applicable (answered with “very often”), the risk of fulfilling five or more out of nine DSM-5 criteria within the subsample of node 5, was 18.4% (see node 5; *n* = 179 IUD-5plus/*n* = 972 subsample node 5). The third best predictive variable to identify those who answered five or more out of nine DSM-5 criteria with “very often” (IUD-5plus) based on the Chi2 values, was “continued overuse.” If the criterion “continued overuse” was applicable (answered with “very often”), the risk of IUD-5plus within the subsample of node 11, was 38.9% (see node 11; *n* = 158 IUD-5plus/*n* = 406 subsample node 11).

The results revealed that 19.9% of node 2 (*n* = 239 IUD-5plus subsample node 2/*n* = 1,199 IUD-5plus entire sample) and 13.2% of node 11 (*n* = 158 IUD-5plus subsample node 11/*n* = 1,199 IUD-5plus entire sample) of all participants who fullfilled five or more of the nine criteria (IUD-5plus) did not answer “jeopardizing” with “very often.”

## 4. Discussion

Which diagnostic criteria distinguish between IUD and non-pathological internet use is highly debated in science and practice (Billieux et al., 2019; Brand et al., 2020; Castro-Calvo et al., 2021). The key finding of the present study is that the criterion “jeopardizing” was found as the best predictor to identify participants who have answered five or more out of nine DSM-5 criteria with “very often” (IUD-5plus), followed by “loss of interest” and “continued overuse.” If these three criteria were applicable, the risk within this subsample to be assigned to IUD-5plus, was 93.6%.

Within the group of all participants who have answered five or more out of nine questions representing the DSM-5 criteria with “very often,” 64.9% were already correctly assigned to IUD-5plus by fulfilling the three DSM-5 criteria based on their questionnaire-based response behavior mentioned above.

The highest endorsement rates in the IUD-4minus, IUD-5plus and entire sample were shown for the criteria “loss of control,” “continued overuse” and “mood regulation.” However, only one of these criteria with the highest endorsement rates was included in the decision tree, which emphasizes that high endorsement of a criterion does not necessarily indicate good diagnostic validity. Especially for the criterion “mood regulation” a high endorsement rate has already been reported in various studies with different samples (Király et al., 2017; Besser et al., 2019; Luo et al., 2021), but it has already been concluded that the criterion “mood regulation” is not well suited to distinguish between pathological and non-pathological internet use (Besser et al., 2019; Castro-Calvo et al., 2021). The reported high endorsement rates for the criteria “loss of control” and “continued overuse” in the present study are in line with studies from Hungarian (Király et al., 2017) and Germany (Besser et al., 2019). In contrast to previous studies (Király et al., 2017; Besser et al., 2019; Luo et al., 2021), the endorsement rate of the criterion “preoccupation” was not among the three highest endorsement rates in the present study. Possible explanations for the different results could be due to various samples and/or different underlying methods to capture the DSM-5 criteria of IGD.

Based on the decision tree analysis, the criterion “jeopardizing” had the highest predictive value to distinguish between participants who fulfilled, based on their questionnaire-based response behavior, the requirements of an IUD following the DSM-5 approach (five and more out of nine criteria were answered with “very often”) and those who did not. The results found are consistent with two clinical studies of patients with IGD (Ko et al., 2014) and IUD (Müller et al., 2019) as well as a questionnaire-based study (Luo et al., 2021). Furthermore an expert panel of practitioners and scientists concluded during a Delphi study that the criterion “jeopardizing” represents a decisive criterion with regard to diagnostic validity, clinical utility and prognostic value (Castro-Calvo et al., 2021). Thus, the results of the present study support the important role of functional impairment and that this criterion is crucial for the diagnosis. If this criterion is not present, no diagnosis should be made to prevent over-pathologization (Billieux et al., 2017; World Health Organization, 2022b).

The second criterion best predicting an IUD based on the questionnaire-based response behavior of the participants is “loss of interest.” The relevance as a predictor for an IUD of this criterion has already been highlighted in studies with large sample sizes of online gamers and/or students in Germany and China (Rehbein et al., 2015; Király et al., 2017; Luo et al., 2021), as well as in clinical studies (Ko et al., 2014; Müller et al., 2014). The expert panel also classified this criterion as relevant in diagnosis, although there was no agreement on inclusion/exclusion with regard to clinical utility and prognostic value (Castro-Calvo et al., 2021).

As already shown in previous studies (Ko et al., 2014; Luo et al., 2021), the criterion “continued overuse” also emerged in the present study as an important predictor to forecast pathological internet users based on their questionnaire-based response behavior following the DSM-5 regulations. The expert panel also rated this criterion as important on all three levels (diagnostic validity, clinical utility, prognostic value) and supported the inclusion of this criterion (Castro-Calvo et al., 2021). In contrast, the study of Király and colleagues showed that “continued overuse” was associated with lower severity of IGD and also in the study of Rehbein and colleagues this criterion was not found to be a decisive predictor (Rehbein et al., 2015; Király et al., 2017). Possible explanations for the different results could be the different operationalization of the diagnostic criteria and/or diversity of samples (i.e., age, gender, IGD vs. IUD).

Overall, all three criteria which were identified within the decision tree analysis as best predictors to forecast IUD based on the questionnaire-based response behavior of the participants following the DSM-5 approach are included not only in the DSM-5 but in the ICD-11 as well (American Psychatric Association, 2013; World Health Organization, 2022b). Additionally, all three criteria are defined as core symptoms of a behavioral addiction (Brand et al., 2020). With regard to the ICD-11 criteria of GD (World Health Organization, 2022b) only the criterion “loss of control” had no high relevance in the present analysis to predict IUD based on the questionnaire-based response behavior of the participants following the DSM-5 approach, even though the expert panel (Castro-Calvo et al., 2021) as well as clinical studies (Ko et al., 2014; Müller et al., 2019) assigned high relevance to this criterion. One hypotheses might be that “loss of control” is a very early feature of IUD (and therefore very sensitive, but not specific), which may develop before other criteria are fulfilled. Therefore, it is important to differentiate between those criteria which seem to be a general warning signal for problematic internet use and those which cover noticeable negative consequences due to internet use (Billieux et al., 2019; Brand et al., 2020).

Thus, the results of the present study support the classification of IUD as behavioral addiction, since core symptoms of addictive behaviors were identified as criteria with the highest predictive power. Therefore, it can be assumed that DSM-5 criteria such as “mood regulation” represent potential processes in the development of behavioral addictions, but these criteria are not suitable to distinguish between IUD and non-pathological behavior (Brand et al., 2020). In others words, the use of the internet for changing mood or alleviating boredom should not be a sign of pathological internet use, but can be an additional clinical feature once the required diagnostic criteria are fulfilled (World Health Organization, 2022b).

The results of the present study emphasize the differences in diagnosing (I)GD depending on the underlying catalog. The DSM-5 regulations are defined by the requirement that arbitrary five or more of the nine DSM-5 criteria need to be fulfilled for an IGD diagnosis (American Psychatric Association, 2013). In contrast, the three ICD-11 criteria and the underlying functional impairment must be present for a diagnosis of GD in ICD-11 (World Health Organization, 2022b). The findings of the present study show that almost 20% of participants who fulfilled five or more out of nine DSM-5 criteria, based on their questionnaire-based response behavior, did not fulfill the criterion “jeopardizing,” which most closely corresponds to the crucial “functional impairment” criterion for diagnosing an GD in ICD-11 (World Health Organization, 2022b). This means that based on the ICD-11 criteria, these individuals would not meet the criteria for a GD diagnosis, even if other relevant criteria such as “loss of interest” and “continued overuse” were fulfilled (answered with “very often”) by the majority of these participants based on their questionnaire-based response behavior. These results indicate that the DSM-5 regulations provide a larger range for different phenotypes including processes underlying the engagement in gaming in early stages of the development of addictive behaviors and core symptoms of GD associated with later stages of the process contributed to the maintenance of addictive behaviors (Brand, 2020). For the more stringent ICD-11 criteria the core symptoms of GD are used as basis to prevent over-pathologization. Therefore, the DSM-5 criteria could be used to determine different stages of addiction development and to relate them to the underlying processes and core symptoms. For the diagnosis of addictive behavior the ICD-11 criteria should be used. Overall, some DSM-5 and almost all ICD-11 criteria seem to be valid to identify IUD based on the questionnaire-based response behavior of the participants. The results support the relevance of the ICD-11 criteria, which may be superior to the DSM-5 criteria in diagnosing individuals with IUD and in preventing false positive diagnoses (Jo et al., 2019). Important to consider when diagnosing IUD are boundaries with other disorders and conditions like disorders due to substance use, bipolar or related disorders (World Health Organization, 2022b).

In addition, the underlying data provides an indication of the particular relevance of the different criteria, which can be used as guidance in screenings and diagnostics. If these results can be replicated in future studies with clinical samples and standardized screenings, this prioritization of diagnostic criteria could be particularly useful for screenings and/or settings in which only a limited amount of time is available for initial assessment. The criterion jeopardizing in particular seems to be the most relevant criterion and should therefore be assessed with particular caution and priority in the diagnostic process. Especially the association to the internet use should always be determined in order to ensure that the harmful behavior results from internet use. Otherwise, potential comorbidities must be identified and taken into account accordingly in the further course of treatment.

In addition to the implications for diagnosing an IUD, such a decision tree can be integrated in the course of treatment. The decision tree can be used during psychoeducation in order to identify individual warning signals together with the patient or within counseling and prevention in order to determine the current internet use behavior considering which criteria are already present.

### 4.1. Limitations

Since the self-test was freely available on the internet, our results are based on a selected sample of people who were interested in receiving feedback regarding their internet use and therefore no conclusions regarding the whole population can be drawn. Clearly more men than women participated in the study and the age range was very broad. To capture the effect of these two variables, those variables were included in the CHAID decision tree analysis. Nevertheless, future studies should pay attention on equal distribution of gender and an evidence-based age range. Due to the intention to reach as many interested persons as possible and to avoid high dropout rates, only the most important questions were collected with regard to the study aim. Further information on internet usage time, specific internet use and/or existing comorbidities should be collected in future studies.

Even though the questionnaire was created following standardized questionnaires and the DSM-5 criteria of IGD, the questionnaire was not validated and therefore first interpretations and directions can be pointed out, but no final conclusions can be drawn. Furthermore, the questionnaire was completed by the participants themselves and there was no structured interview to capture the diagnostic criteria in a third party rating. To strengthen the validity of the results found in this study, future studies should use standardized questionnaires and structured interviews for diagnosis.

## 5. Conclusion

The main finding of the present study is that the criterion “jeopardizing,” “loss of interest” and “continued overuse” best predict participants who fulfilled based on their questionnaire-based response behavior the requirements of an IUD following the DSM-5 approach (five and more out of nine criteria were answered with “very often”), which is widely consistent with the ICD-11 criteria of GD and therefore the use of ICD-11 criteria should be the better option to prevent over-pathologization. One particular topic for future studies may be to apply the DSM-5 diagnostic criteria to specific stages of IUD (e.g., risky versus pathological use) since diminished control, priority, and continuation may be differently related to specific driving paths to addiction and reduced self-control (Brand et al., 2020).

## Data availability statement

The original contributions presented in the study are included in the article/Supplementary material, further inquiries can be directed to the corresponding author.

## Ethics statement

The studies involving human participants were reviewed and approved by Ruhr University Bochum. The patients/participants provided their written informed consent to participate in this study.

## Author contributions

LB conducted literature research, created manuscript concept, ran statistical analyses, interpreted the results, and wrote the manuscript. MB and BW contributed to the manuscript concept and refinements. BW conceived the study and acquired funding. LB, BW, and JD-H conducted and coordinated the study. MB, BW, JD-H, SH, and MP contributed to the style of reporting and writing and approved the final version of the manuscript.

## Funding

This publication was funded by the German Federal Ministry of Health under grant number ZMVI1-2516DSM207.

## Conflict of interest

The authors declare that this study received funding from the German Federal Ministry of Health under grant number ZMVI1-2516DSM207. The study design was approved by the funder. The funder supported recruitment by drawing attention to the project. The funder was not involved in the study analysis, interpretation of data, the writing of this article or the decision to submit it for publication.

## Publisher’s note

All claims expressed in this article are solely those of the authors and do not necessarily represent those of their affiliated organizations, or those of the publisher, the editors and the reviewers. Any product that may be evaluated in this article, or claim that may be made by its manufacturer, is not guaranteed or endorsed by the publisher.
