# Supplementary material for: Predictive power of the DSM-5 criteria for internet use disorder: A CHAID decision-tree analysis
Source: Front Psychol. 2023 Feb 22;14:1129769. doi: 10.3389/fpsyg.2023.1129769 (PMC9994355; doi:10.3389/fpsyg.2023.1129769)
Supplement: Supplementary file 1 [file Table_1.docx]

Supplementary Material

Predictive power of the DSM-5 criteria for internet use disorder: A CHAID decision-tree analysis

**Laura Bottel*, Matthias Brand, Jan Dieris-Hirche, Magdalena Pape, Stephan Herpertz, Bert Theodor te Wildt**

*** Correspondence:** Laura Bottel, laura.bottel@ruhr-uni-bochum.de

# Supplementary Table

**Table 3:** Listing of the nine DSM-5 criteria of Internet Gaming Disorder (IGD; American Psychatric Association, 2013), the developed questionnaire under consideration of the DSM-5 diagnostic criteria of IGD (American Psychatric Association, 2013) and according to the short-form scale to assess Internet Gaming Disorder (IGDS-SF9; Pontes and Griffiths, 2015) and the English translation of the developed questionnaire. In line with the IGDS-SF9 the nine questions were answered on a 5-point Likert scale from “never” to “very often”.

| **DSM-5 criteria of IGD** (American Psychatric Association, 2013) | **German version** of developed questionnaire | **English translation** of German version of developed questionnaire |
| --- | --- | --- |
| (1) preoccupation with internet games | Verbringen Sie viel Zeit damit an Aktivitäten im Internet zu denken, auch wenn Sie gerade nicht online sein können, oder damit zu planen, wann Sie wieder ins Internet gehen wollen? | Do you spend a lot of time thinking about activities on the internet, even when you can't be online at the moment, or planning when you will go online again? |
| (2) withdrawal symptoms when internet gaming is taken away | Fühlen Sie sich ruhelos, gereizt, launisch, wütend, ängstlich oder traurig, wenn Sie versuchen weniger oder gar nicht online zu sein oder wenn Sie keine Möglichkeit haben ins Internet zu gehen? | Do you feel restless, irritable, moody, angry, anxious, or sad when you try to be online less or not at all, or when you don't have a chance to go online? |
| (3) tolerance, resulting in the need to spend increasing amounts of time engaged in internet games | Brachen Sie längere Zeiten im Internet, aufregendere Internetaktivitäten oder bessere Geräte, um das gleiche Ausmaß an Spannung wie früher zu erreichen? | Do you need increasing amounts of time on the internet, more exciting internet activities, or better devices to achieve the same level of excitement as before? |
| (4) unsuccessful attempts to control participation in internet games | Haben Sie das Gefühl, dass Sie weniger online sein sollten, schaffen es aber nicht ihre Zeiten im Internet zu verringern? | Do you feel like you should be online less, but can't manage to reduce your times on the internet? |
| (5) loss of interest in previous hobbies and entertainment as a result of, and with the exception of, internet games | Verlieren Sie wegen Ihrer Internetaktivitäten das Interesse an anderen Freizeitaktivitäten (Hobbys, Freunde) oder schränken Sie diese ein? | Do you lose interest in or limit previous hobbies and entertainment as a result of your internet activities? |
| (6) continued excessive use of internet games despite knowledge of psychosocial problems | Setzen Sie Ihre Internetaktivitäten in gleichem Ausmaß fort, obwohl Sie sich der negativen Folgen bewusst sind, wie Schlafmangel, Unpünktlichkeit in Schule/Arbeit, zu hohe Geldausgaben, Streitigkeiten mit anderen, oder Vernachlässigung wichtiger Pflichten? | Do you continue your internet activities at the same rate despite your knowledge of psychosocial problems, such as lack of sleep, late arrival at school/work, spending too much money, arguments with others, or neglect of important duties? |
| (7) deceiving family members, therapists, or others regarding the amount of internet gaming | Belügen Sie Familienmitglieder, Freunde oder andere über das Ausmaß Ihrer Internetaktivitäten oder versuchen Sie Ihre Online-Zeiten vor Familienmitgliedern oder Freunden zu verheimlichen? | Do you deceive family members, friends, or others about the extent of your internet activity, or try to hide your online time from family members or friends? |
| (8) use of internet games to escape or relieve negative moods; (e.g. feelings of helplessness, guilt, anxiety) | Nutzen Sie das Internet um persönlichen Problemen zu entkommen oder um diese zu vergessen, oder um unangenehme Gefühle wie Schuld, Angst, Hilflosigkeit oder Niedergeschlagenheit zu lindern? | Do you use the internet to escape or forget personal problems, or to relieve negative moods such as guilt, anxiety, helplessness or dejection? |
| (9) jeopardizing or losing a significant relationship, job, or education or career opportunity because of participation in internet games | Gefährden oder verspielen Sie wegen Ihrer Internetaktivitäten wichtige Beziehungen, Ihre Arbeitsstelle oder Möglichkeiten in Bildung und Beruf? | Are you jeopardizing or losing significant relationships, your job, or educational and career opportunities because of your participation in internet activities? |
